# The impact of bias of underlying literature in guidelines on its recommendations: assessment of the German fluoride guideline

**DOI:** 10.1007/s40368-023-00854-7

**Published:** 2023-11-26

**Authors:** A. Al Masri, U. Schiffner, M. S. Mourad, J. Schmoeckel, P. Joseph, C. H. Splieth

**Affiliations:** 1 Department of Preventive and Pediatric Dentistry, Greifswald University Dental Clinics, Walther-Rathenau-Straße 42a, 17475 Greifswald, Germany; 2 Department for Periodontology, Preventive and Restorative Dentistry, Center for Dental and Oral Medicine, University Medical Center Hamburg-Eppendorf (UKE), Martinistraße 52, 20246 Hamburg, Germany; 3 Department of Orthodontics, Greifswald University Dental Clinics, Walther-Rathenau-Straße 42a, 17475 Greifswald, Germany

**Keywords:** Bias, Guideline, Fluoride, Caries prevention, Paediatric dentistry, Children

## Abstract

**Purpose:**

The significance of the underlying literature in clinical guidelines can be weakened by the risk of bias, which could negatively affect the recommendations. Especially in controversial matters, such as fluoride use for caries prevention in children, biased results may be not reliable and lead to incorrect conclusions. This study was performed to detect bias in underlying literature of the German guideline for caries prevention using fluoride in children, where no consensus was reached between paediatricians and paediatric dentists.

**Methods:**

Three tools used for risk of bias assessments of different study designs were RoB 2 for RCTs, ROBINS-I for non-randomized studies, and ROBIS for systematic reviews. For each study cited in the guideline two independent risk of bias assessments were performed. Disagreements were resolved by consensus.

**Results:**

Out of 58 papers, 48.3% (*n* = 28) showed high risk of bias, with the majority in sections regarding fluoride tablets, fluoridated toothpaste, and paediatricians’ recommendations. 9 out of 20 recommendations and statements were based on studies with high risk of bias, all of which were in these three controversial sections. 13 out of 29 RCTs showed high risk of bias (44.8%), as all 13 non-randomized trials did, while only 2 of 16 (12.5%) systematic reviews had high risk of bias.

**Conclusion:**

Considering risk of bias of cited studies in clinical guidelines may result in substantial changes in its recommendations and aid in reaching consensus. Efforts should be made to assess risk of bias of underlying literature in future clinical guidelines.

**Supplementary Information:**

The online version contains supplementary material available at 10.1007/s40368-023-00854-7.

## Introduction

Clinical decision making is becoming more dependent on evidence-based systematic reviews and scientific guidelines with clinical recommendations (Hakkennes and Dodd 2008; Kranke 2010; Medves et al. 2010), because they make a significant contribution to the transfer of scientific knowledge to the clinical practice with ratings of the underlying literature depending on the quality and certainty of the evidence (Hakkennes and Dodd 2008). The implementation of guidelines in the clinical practice positively affects the medical care providers as well as the patients and strengthens the trust in this relationship (Medves et al. 2010). Guidelines providing clinical recommendations could, therefore, solve many controversies in the medical field, as they are or at least should be based on solid evidence with high quality. Nevertheless, in many health-related issues, a wide variety of papers regarding the same matter and having the same PICO/PECO framework (Population, Intervention/Exposure, Comparator, and Outcome) may come to different conclusions. This may confuse the clinical practitioner, and make the mission of developing guidelines more challenging. For example, despite the worldwide agreement in the latest recommendations and systematic reviews on the effectiveness of fluoride in caries prevention as well as in the arrestment of active caries lesions (Walsh et al. 2019; AAPD 2020; PHE 2017), there are still knowledge gaps regarding the appropriate amount and concentration of fluoride for caries prevention by preschool children as concluded in the EAPD guidelines from 2019 (Toumba et al. 2019). This may be due to the huge variety of fluoride-containing products with different concentrations, all of which are supported by evidence, which makes an international agreement in the light of the underlying evidence not always possible. Not only on the international level, but in some countries, agreements on this matter are difficult to reach on the national level. In Germany, for example, in the latest guidelines for the use of fluoride in caries prevention in children in 2013 (Hellwig et al. 2013) no consensus could be obtained between paediatricians’ and dentists’ recommendations regarding the optimal method for the application of fluoride for caries prevention in early childhood (Mourad et al. 2018; DAKJ 2007). In these guidelines, the following sentence was stated in an extra section reporting the consensus between dentists and paediatricians: ‘‘In the coordination of the present guidelines, divergent recommendations were concluded between the representatives of dentists and paediatricians in a formal consensus process resulting in no consensus regarding the recommendation for the use of fluoridated toothpaste and/or fluoride tablets at preschool age’’. Since each recommendation from both communities is supported by some evidence, a consensus would not be possible without critical assessments of the underlying literature regarding quality level and risk of bias. Obviously biased results (whether on purpose or due to poor presentation of study design or results) could be false positive or even false negative, which may explain the variation in the results of studies with similar PICO/PECO framework (Higgins et al. 2020). Considering that no agreement in the consensus was reached, the underlying literature should be critically reviewed, because many cited papers used as evidence could be of low quality, low evidence-base or have a high risk of bias. The latter is to be discussed in this review on the risk of bias, because it was not included in the process of guidelines’ development. An elimination or at least a cautious approach when citing studies with high risk of bias may lead to different outcomes and avoid disagreements between experts.

Thus, the aim of this study is to assess risk of bias in all cited references from the 2013 German guidelines for the use of fluoride in caries prevention in children using available risk of bias assessment tools to find out the potential impact of high risk of bias of evidence on the clinical recommendations, which may aid in solving controversies regarding the most effective methods and concentrations of fluoride use in caries prevention in children. This could dramatically change the outcome and the strengths of the recommendations in this clinical guideline.

## Materials and methods

All the studies cited (*n* = 80) in the latest German guideline for the use of fluorides in caries prevention in children (Hellwig et al. 2013) were included in this review.

The studies were obtained in full version, and authors of non-available studies were contacted and asked to provide a full version of their publication. The papers were allocated to four categories according to the type of each study (see Table 1):

- Category 1: RCTs (Randomized Clinical Trials)
- Category 2: Non-randomized study designs
- Category 3: Systematic reviews
- Category 4: Other papers (reviews, guidelines with no detailed methodology, reports, etc.)

**Table 1:** Overall risk of bias in the included papers according to study design and the used tool (*n* = 58)

Study design	Used tool	Overall risk of bias assessment	% (n)
Randomized clinical trials (*n* = 29)	RoB 2	High	44.8% (13)
		Moderate	27.6% (8)
		Low	27.6% (8)
Non-randomized trials (*n* = 13)	ROBINS-I	High	100% (13)
		Moderate	0
		Low	0
Systematic reviews (*n* = 16)	ROBIS	High	12.5% (2)
		Moderate	18.8% (3)
		Low	68.8% (11)
Total number of assessed papers (*n* = 58)	RoB2 ROBINS-I ROBIS	High	48.3% (28)
		Moderate	19% (11)
		Low	32.7% (19)

Non-systematic reviews and guidelines with no detailed methodology cannot be assessed regarding risk of bias, and the fourth group was, therefore, excluded from this assessment. In total, 22 papers were excluded due to different reasons, a list of the papers and reasons for their exclusion are reported in appendix 1.

The methodological quality of the included studies in terms of evidence level was discussed in the guideline, with assessments of evidence level according to the study design as well as a critical appraisal. However, these do not include the detection and assessment of risk of bias, as this aspect was probably not considered relevant when the guideline was developed in 2013. With more focus on risk of bias recently in medical research, special risk of bias assessment tools are developed and increasingly used in the literature, which are needed for the assessments in this review. Since no tool could be used to assess the risk of bias in all study types, different tools were needed to appropriately assess the various studies in the different categories. The choice for the appropriate tool should be made carefully and the assessments should be done at least twice by two independent assessors to ensure reliable assessments with no performance bias (Ma et al. 2020).

Three risk of bias assessment tools that are similar in their structures were identified and used according to the guidance of their authors:

- RoB 2: “Revised Cochrane risk-of-bias tool for randomized trials’’ for RCTs (Higgins et al. 2016, 2018),
- ROBINS: “Risk of Bias In Non-randomized Studies’’ for studies with non-randomized design (Sterne et al. 2016a, b), and
- ROBIS: ‘‘Risk of Bias in Systematic reviews’’ for systematic reviews (Whiting et al. 2016).

For each tool, the developers provide blank templates and a detailed guidance that are to be used to follow the instructions of the developers. For each included study, a blank template of the appropriate tool was filled out by each assessor and an overall risk of bias assessment was afterwards reached. The detailed description of the methodology of each tool can be obtained from the official website of the developers of the tools or from their publications (Higgins et al. 2016, 2018; Sterne et al. 2016a, b; Whiting et al. 2016). The three used tools have similar structures based on questions in different aspects, as can be seen in modified summaries of the tools in appendix 2. In general, for each study design, there are main domains to be assessed, which correspond to the main possible sources of risk of bias for each study type. Many questions for each domain should be answered and supported with evidence from the study being assessed to reach an assessment of risk of bias for this domain. The overall risk of bias judgement for each study can be reached after risk of bias assessments of all domains. For a study to be assessed with low risk of bias, all the domains should show low risk of bias. On the other hand, one domain with high risk of bias would put the paper directly in a high risk of bias for the overall assessment. While the RoB 2 and the ROBIS tools have very similar outcome assessments of risk of bias for each study (high, some concerns/moderate/unclear, or low), the ROBINS-I tool uses more detailed classification for risk of bias assessments in non-randomized trials (critical, serious, moderate, low, or no information). For the purpose of this study, and to allow for better reporting of our results as well as for a comparison between risk of bias in the included studies with different types, the risk of bias assessments for the ROBINS-I tool were modified to have only three outcome possibilities such as the other two tools considering ‘‘critical’’ and ‘‘serious’’ as ‘‘’high’’. This modification was done after completing the assessment of each study, so that the tools were used in their original versions. The three possible assessments for the pooled papers were therefore ‘‘High’’ including (critical and serious), ‘‘Moderate’’ including (some concerns, unclear, and no information), or ‘‘Low’’.

A total of four researchers performed the risk of bias assessments, each study was assessed two times independently by two different researchers (AAM and MSM for RCTs, AAM and JS for non-randomized trials, as well as AAM and PJ for systematic reviews). Disagreements were solved by the assessors to reach consensus through discussion and revision. A third researcher (SP) was available in case of disagreements after the discussion. The filled templates for each study may be requested by the corresponding author of this review.

Risk of bias in the multiple domains from each paper and the overall risk of bias assessments of all included papers from all assessors were transferred into Excel sheets (Microsoft Office^®^) for descriptive statistical analysis.

Furthermore, the conclusions of the included studies were summarized. The recommendations and statements of the guidelines were collected and assessed regarding risk of bias of the underlying evidence.

## Results

**Fig. 1:**
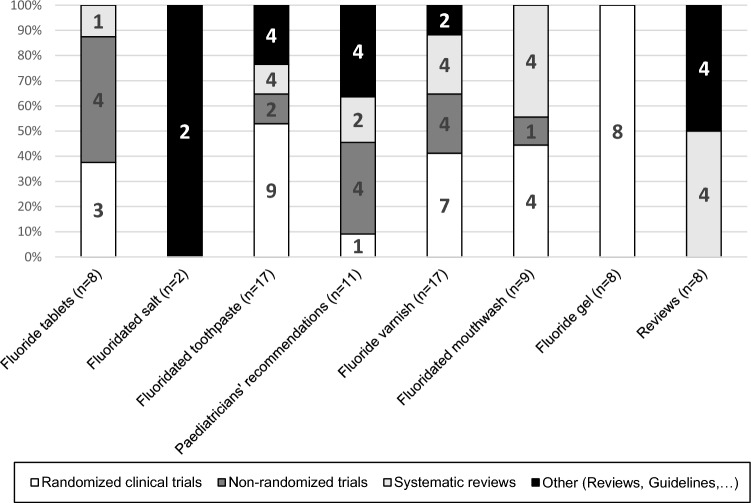
Distribution and percentage of cited papers in the different sections of the German fluoride guidelines (2013) categorized according to study type (*n* = 80)

The latest German Guidelines for the use of fluorides in caries prevention divided the cited papers according to the method of fluoride administration in six main sections with two additional sections: (1) Papers that were used by the paediatricians in their recommendations and in a consensus with the dentists in 2012 and (2) Overview papers for the effect and safety of fluoride (Hellwig et al. 2013). Figure 1 illustrates the total number of papers and the percentage of study types within each section. Some sections such as ‘‘Fluoride gels’’ cited mostly studies with high evidence level such as RCTs, while in other sections such as ‘‘fluoridated salts’’ only reports and non-systematic reviews were cited.

Non-available studies, multiple papers of one trial, non-systematic reviews and guidelines or recommendations with no clear methodology cannot be assessed regarding risk of bias and were thus excluded (appendix 1). Therefore, 58 out of 80 cited papers remained for risk of bias assessment using the mentioned tools (RoB 2, ROBINS-I, ROBIS) according to the corresponding study type.

The total number of the included RCTs was 29, of which 44.8% (*n* = 13) showed high risk of bias. 13 non-randomized trials were included in the assessments, with none of them having a low or moderate risk of bias. Using the ROBIS tool, 68.8% of the included systematic reviews (*n* = 16) were assessed with a low risk of bias. Table 1 shows the overall assessments of risk of bias for all included studies according to their study design (RCTs, non-randomized trials, or systematic reviews).

**Fig. 2:**
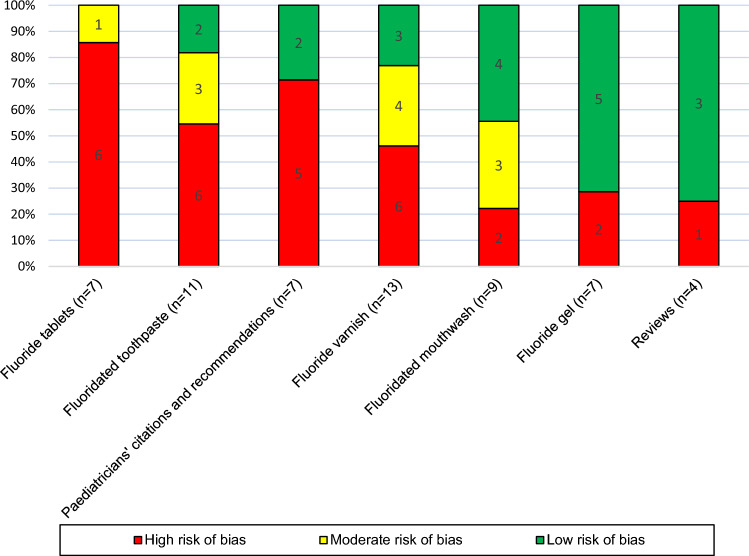
Overall risk of bias assessments in the papers cited in the German fluoride guideline’s sections regardless of study design and the used tool (*n* = 58)

Figure 2 shows the risk of bias assessments of the papers according to the section in the guidelines, in which they were cited regardless of their study design. The controversial sections of the guidelines regarding toothpaste and fluoride tablets as well as the paediatricians’ recommendations showed higher percentage of underlying literature with high risk of bias. No section was based completely on papers with low and moderate risk of bias.

A detailed overview of overall risk of bias assessments of all included papers in the different sections of the guidelines in all study designs as well as the main conclusion of each paper is available in appendix 3.

**Fig. 3:**
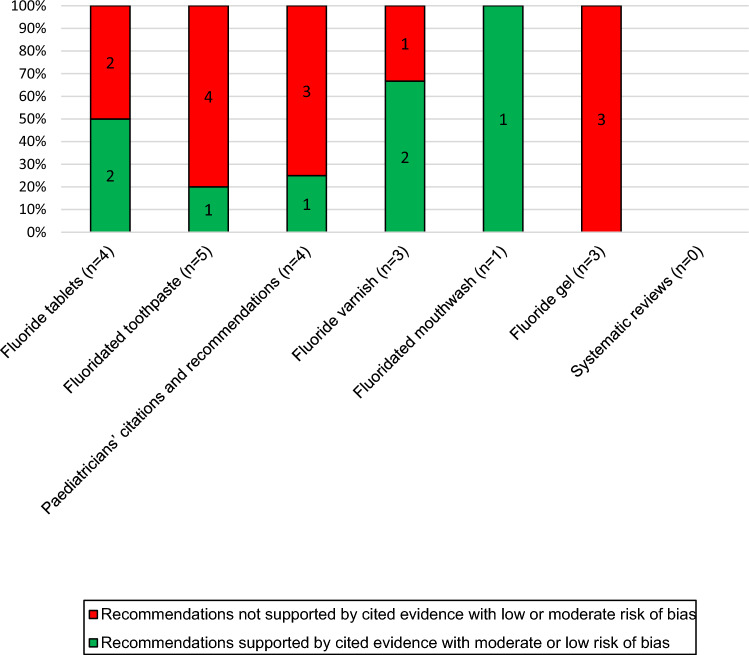
Assessment of the recommendations of the German fluoride guideline considering risk of bias of underlying literature in the different sections (*n* = 20)

All the statements and recommendations of the assessed guideline were collected in a table (*n* = 20). For each statement, the underlying evidence was identified if possible and reviewed to check its risk of bias (appendix 4). More than half of the recommendations were not supported by evidence with low or moderate risk of bias (*n* = 13), the majority of which were in the sections regarding fluoride tablets, fluoridated toothpaste, paediatricians’ recommendations and fluoride gel (Fig. 3).

## Discussion

Controversies and disagreements may arise between dental and medical communities or between different areas of the same medical specialty. This may be particularly the case when the statements presented are supported by evidence but have not been evaluated for quality and risk of bias. In the earlier mentioned controversy in Germany, the paediatricians provided evidence for the high efficacy of fluoride tablets in caries prophylaxis and recommended their use in early childhood, while the paediatric dentists recommended low concentrated fluoridated toothpaste instead of tablets and presented evidence to support their recommendations. Therefore, despite having evidence background for each recommendation, the reliability of the underlying literature should be thoroughly assessed to reach consensus. Although the level of evidence is essential to assess the reliability of results of studies, but it is not sufficient, because an RCT or a systematic review of RCTs has a high evidence level, but might have high risk of bias, which is a systematic error or deviation from the truth that may distort the results (Higgins et al. 2020). A correct evaluation of the risk of bias can signalize the reliability of the results, which may improve the quality of dental treatments (Faggion 2015). The evaluation of the risk of bias in the evidence underlying the recommendations is one of the factors, that influences the strengths of recommendations and was performed in this study.

As recommended by the Cochrane Collaboration, tools for Risk of Bias assessment should not use scales and scores and may rather use the terms “low risk”, “high risk”'', or “unclear risk”' for the assessment (Lundh and Gotzsche 2008; Higgins et al. 2020). The three tools used in this study fulfilled this criterion. (1) The first tool was RoB 2, which is a Revised Cochrane risk-of-bias tool for randomized trials. The tool was found to be quite simple and easy to use with its detailed guidance. (2) The ROBINS-I tool was the second tool, which was developed by members of the Cochrane Bias Methods Group and the Cochrane Non-Randomized Studies of Interventions Methods Group and is based on the concepts of the Cochrane RoB tool for randomized trials. Although the ROBINS-I tool provides a useful method to assess the risk of bias in non-randomized trials, many reports have criticized it because of the difficulty in its application, the weak guidance, and poor reliability (Sun et al. 2018; Thomson et al. 2018; Minozzi et al. 2019). However, the ROBINS-I tool provides a substantial improvement over the formerly widely used Newcastle–Ottawa Scale, which has also been sharply criticized (Stang 2010; Huffman and Thomas 2018). A modification on this tool called ROBINS-E for trials of exposure is developed and newly updated (Morgan et al. 2019), but it was at the time of assessments in this study only a preliminary tool and was criticized for not meeting international standards for evaluating human observational studies (Bero et al. 2018). In comparison to other available tools for risk of bias assessment of non-randomized trials, the use of ROBINS-I might be acceptable (Schünemann et al. 2019). (3) The third tool (ROBIS) is reported to be the first tool that is specially designed for the risk of bias assessment of systematic reviews (Hu et al. 2018). Moreover, this tool is recently increasingly used in the literature and as found in our study is reported to be reliable and easy to use (Buhn et al. 2017).

It is noteworthy that although all the assessed studies are published before the development of the used risk of bias assessment tools, the studies varied from low to high risk of bias in the assessments. This shows that also before the recently increased attention to risk of bias, well performed older studies managed to control the risk of bias without having these modern assessment tools to aid them in avoiding bias. Future studies should use the opportunity of having such tools to guide them in minimizing the risk of bias in the planning stages.

Unfortunately, but not surprisingly, 44.8% of the assessed RCTs showed high risk of bias (*n* = 13). Previous studies assessing risk of bias even in RCTs mostly reported similar results (Papageorgiou et al. 2015; Elangovan et al. 2016). This underlines the necessity of improving the quality of study design and reporting of RCTs because biased results of RCTs tend to increase the effect size estimation and raise suspicion about their reliability (Page et al. 2016; Saltaji et al. 2018).

None of the included non-randomized trials was assessed to have low or moderate risk of bias. This is mostly due to the difficulty of avoiding risk of bias in such study designs in general. While a randomized study design should be considered for proving the efficiency of therapeutic and preventive clinical measurements, it is in many cases not possible to randomize the participants, especially in matters such as the caries prevention effect of fluoridated salts or fluoridated water supply. Therefore, when it is not avoidable to include non-randomized trials in systematic reviews or in clinical guidelines and recommendations, the included trials should be assessed for risk of bias, and their results should be interpreted with caution (Schünemann et al. 2019).

Systematic reviews are mostly considered of high level in the medical field. However, this should not be taken for granted, as there are many components, that affect the quality and the reliability of systematic reviews. Risk of bias of systematic reviews is one of these components and should always be considered when interpreting the results of a systematic review. In this review, 68.8% of the included systematic reviews were assessed with a low risk of bias (*n* = 11), including all the six assessed “’Cochrane reviews’’, which agrees with recent systematic and umbrella reviews, where all the included “’Cochrane reviews’’ had always low risk of bias compared to other systematic reviews (Faggion et al. 2015; BaniHani et al. 2021). Two systematic reviews did not report the specific search methods or an overview of the included studies in detail and had, therefore, a high risk of bias.

The findings of the overall risk of bias assessments of the cited papers in the 2013 German guidelines for the use of fluoride in caries prevention showed a high risk of bias for nearly half of the papers regardless of their study design (48.3%; *n* = 28). This result agrees with a previous review, that reported a high percentage of risk of bias in cited papers of the American Heart Association guidelines (Cho et al. 2019). Authors of clinical trials and systematic reviews as well as clinical guidelines should be therefore encouraged to consider risk of bias during planning to ensure a good reliability of their results and to raise the quality of evidence to a trustworthy level (Bradley et al. 2020).

Regarding risk of bias in the papers cited in different sections of the German fluoride guidelines, it was found that the percentage of cited papers having a low or moderate risk of bias was high in the sections concerning fluoridated mouthwash and fluoridated gel. On the other hand, the sections regarding fluoride tablets, fluoridated toothpastes, and the paediatrician’s recommendations were based on papers, from which more than 50% had a high risk of bias (85.7%, 54.5%, and 71.4% respectively). This could explain the failure to reach consensus regarding the use of fluoride for caries prevention in early childhood, where the paediatricians recommended the use of fluoride tablets or lozenges, while the paediatric dentists recommended the use of low concentrated fluoridated toothpaste starting with the eruption of the first primary tooth. A further look in the recommendations of the guidelines showed that 13 out of 20 recommendations and statements could not be supported with evidence of low or moderate risk of bias, many of which were in these three controversial sections. Considering risk of bias of underlying literature in forming the recommendations would probably change the outcome or the strengths of recommendations, which results in avoidance of confusion for the clinicians or the patients, due to discrepancies in the statements.

This study, as any other study has its limitations, which should be considered when interpreting the results; 22 cited papers were excluded from risk of bias assessment due to different reasons, which are mentioned in appendix 1. The exclusion of four studies, where the full text was not found might have an effect on the overall assessment of the recommendations. As these studies are almost equally distributed between the sections of the guidelines, the impact of the exclusion on the overall results and conclusions of this study is thought to be minimal and rather neutral, but could still be possible. Moreover, although the assessors had sufficient time and experience to understand and train on the use of the risk of bias assessment tools, a formal training or piloting was not performed, and should be considered in future similar studies. However, all assessors are members of teaching staff of universities and have the knowledge as well as the ability to perform such assessments. Moreover, the fact, that a total of four researchers participated in the assessments of risk of bias, should eliminate any performance bias or personal preferences in the assessments.

As the results of this review show, clinical guidelines should not be taken for granted, without a critical consideration of their methodology. It is essential to assess the risk of bias of cited papers before providing updates on clinical recommendations and guidelines that would likely affect clinical decisions of practitioners. It may be, therefore, advantageous to follow the AGREE checklist (Brouwers et al. 2016) and/or the GRADE methodology (Guyatt et al. 2008) in the process of guideline development, as the EAPD guidelines for the use of Fluoride in caries prevention did, where not only GRADE ratings were used, but also the recommendations were categorised in STRONG and CONDITIONAL for patients, clinicians and policy makers (Toumba et al. 2019). This will ensure a sound methodology and deliver clear recommendations without dropping any major aspects such as risk of bias assessments. Using specially developed digital software for this purpose such as GRADEpro (GDT 2022) or MAGICAPP (2022) may also increase the transparency during development to have guidelines with high quality and trustworthy recommendations.

It is noteworthy to state, that within the preparation of this paper, a consensus in Germany was announced and a new unified recommendation from the paediatricians and the dentists regarding caries prevention in early childhood was published (Berg et al. 2021).

## Conclusion

The results of this review confirm, that recommendations of guidelines may be strongly affected by risk of bias of the underlying literature and, therefore, emphasize the necessity for risk of bias assessments of citations in future guidelines to provide the clinical practitioners with trustworthy clinical recommendations.

### Supplementary Information

Below is the link to the electronic supplementary material.

Supplementary file1 (DOCX 716 kb)
