# Supplementary material for: The impact of bias of underlying literature in guidelines on its recommendations: assessment of the German fluoride guideline
Source: Eur Arch Paediatr Dent. 2023 Nov 26;25(1):65–73. doi: 10.1007/s40368-023-00854-7 (PMC10942900; doi:10.1007/s40368-023-00854-7)
Supplement: Supplementary file 1 — Supplementary file1 (DOCX 716 kb) [file 40368_2023_854_MOESM1_ESM.docx]

# **Appendices**

**Appendix 1: Excluded papers and the reasons for exclusion (n=22)**

| Cited reference | Reason for exclusion |
| --- | --- |
| - Schulte 2005 - Marthaler et al. 2005 - Buzalaf et al. 2011 - Chu and Lo 2005 - Oganessian et al. 2007 - Marthaler and Pollak 2005 | Non-systematic reviews |
| - Kallestal et al., 2007 - Kallestal et al., 2005 - Pieper et al., 2007 | Different papers of one included trial |
| - Andersson et al., 1976 - Ferreira et al., 2005 - Steiner et al., 2004 - Türksel Dülgergil et al., 2005 | No full text available |
| - Adair 2006 - Arnadottir et al. 2004 - Petersen and Lennon 2004 - American academy of pediatrics committee on nutrition 1995 - Deutsche Akademie für Kinder- und Jugendmedizin 2007 - AAPD Guidelines on Fluoride Therapy 2008 - American Dietetic Association 2005 - Scottish Intercollegiate Guidelines Network (SIGN) 2005 - Autio-Gold 2008 | Guidelines, recommendations, or conference papers with no detailed methodology |

*References of appendix 1 (in alphabetical order):

- Adair SM. Evidence-based use of fluoride in contemporary pediatric dental practice. Pediatr Dent. 2006;28(2):133-42; discussion 92-8.
- American Academy of Pediatrics Committee on Nutrition. Fluoride supplementation for children: interim policy recommendations. Pediatrics. 1995;95(5):777.
- American Academy on Pediatric Dentistry (AAPD). Liaison with Other Groups C, American Academy on Pediatric Dentistry Council on Clinical A. Guideline on fluoride therapy. Pediatr Dent. 2008;30(7 Suppl):121-4.
- American Dietetic Association. Palmer C, Wolfe SH. Position of the American Dietetic Association: the impact of fluoride on health. J Am Diet Assoc. 2005;105(10):1620-8. doi: 10.1016/j.jada.2005.08.017.
- Andersson R, Grahnen H. Fluoride tablets in pre-school-age--effect on primary and permanent teeth. Sven Tandlak Tidskr. 1976;69(5):137-43.
- Arnadottir IB, Ketley CE, Van Loveren C, Seppa L, Cochran JA, Polido M, et al. A European perspective on fluoride use in seven countries. Community Dent Oral Epidemiol. 2004;32 Suppl 1:69-73. doi: 10.1111/j.1600-0528.2004.00142x.
- Autio-Gold J. Recommendations for fluoride varnish use in caries management. Dent Today. 2008;27(1):64-7; quiz 7, 58.
- Buzalaf MAR, Pessan JP, Honorio HM, Ten Cate JM. Mechanisms of action of fluoride for caries control. Monogr Oral Sci. 2011;22:97-114. doi: 10.1159/000325151
- Chu CH, Lo EC. A review of sodium fluoride varnish. Gen Dent. 2006;54(4):247-53.
- DAKJ (Deutsche Akademie für Kinder- und Jugendmedizin): der weitgehenden Vermeidbarkeit W. zur Prävention der Milchzahnkaries*.* Monatsschr Kinderheilkd. 2007;155:544-8.
- Dulgergil CT, Ercan E, Yildirim I. A combined application of ART-fluoride varnish for immigrant junior field-workers: 12-months follow-up field trial in rural Anatolia. Oral Health Prev Dent. 2005;3(2):97-104.
- Ferreira MA, Latorre Mdo R, Rodrigues CS, Lima KC. Effect of regular fluoride gel application on incipient carious lesions. Oral Health Prev Dent. 2005;3(3):141-9.
- Kallestal C, Fjelddahl A. A four-year cohort study of caries and its risk factors in adolescents with high and low risk at baseline. Swed Dent J. 2007;31(1):11-25.
- Kallestal C. The effect of five years' implementation of caries-preventive methods in Swedish high-risk adolescents. Caries Res. 2005;39(1):20-6. doi: 10.1159/000081652.
- Marthaler TM, Petersen PE. Salt fluoridation--an alternative in automatic prevention of dental caries. Int Dent J. 2005;55(6):351-8. doi: 10.1111/j.1875-595x.2005.tb00045.x.
- Marthaler TM, Pollak GW. Salt fluoridation in Central and Eastern Europe. Schweiz Monatsschr Zahnmed. 2005;115(8):670-4.
- Oganessian E, Lencova E, Broukal Z. Is systemic fluoride supplementation for dental caries prevention in children still justifiable? Prague Med Rep. 2007;108(4):306-14.
- Petersen PE, Lennon MA. Effective use of fluorides for the prevention of dental caries in the 21st century: the WHO approach. Community Dent Oral Epidemiol. 2004;32(5):319-21. doi: 10.1111/j.1600-0528.2004.00175.x.
- Pieper K, Born C, Hartmann T, Heinzel-Gutenbrunner M, Jablonski-Momeni A. Association of preventive measures with caries experience expressed by outcome variables. Schweiz Monatsschr Zahnmed. 2007;117(10):1038-44.
- Schulte AG. Salt fluoridation in Germany since 1991. Schweiz Monatsschr Zahnmed. 2005;115(8):659-62.
- Scottish Intercollegiate Guidelines Network (SIGN): Prevention and management of dental decay in the pre-school child. A national clinical guideline. Scottish Intercollegiate Guidelines Network (SIGN): 2005; 41.
- Steiner M, Helfenstein U, Menghini G. Effect of 1000 ppm relative to 250 ppm fluoride toothpaste. A meta-analysis. Am J Dent. 2004;17(2):85-8.

**Appendix 2: Modified summaries of the used risk of bias assessment tools**

**Appendix 2a. Summary of the RoB 2 tool [Higgins et al., 2016].**

**Domain 1: Risk of bias arising from the randomization process**1.1 Was the allocation sequence random?

1.2 Was the allocation sequence concealed until participants were enrolled and assigned to interventions?

1.3 Did baseline differences between intervention groups suggest a problem with the randomization process?


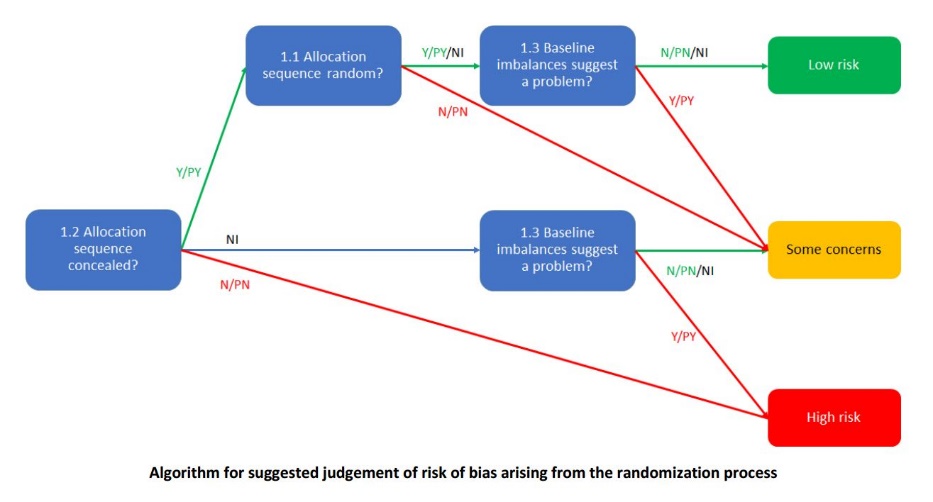


**Domain 2 (a): Risk of bias due to deviations from the intended interventions (effect of
assignment to intervention)**2.1 Were participants aware of their assigned intervention during the trial?
2.2 Were carers and people delivering the interventions aware of participants' assigned intervention
during the trial?
2.3 If Y/PY/NI to 2.1 or 2.2: Were there deviations from intended intervention that arose due to the experimental context?
2.4 If Y/PY to 2.3: Were these deviations from intended intervention balanced between groups?
2.5 If N/PN/NI to 2.4: Were these deviations likely to have affected the outcome?
2.6 Was an appropriate analysis used to estimate the effect of assignment to intervention?
2.7 If N/PN/NI to 2.6: Was there potential for a substantial impact (on the result) of the failure to analyse participants in the group to which they were randomized?


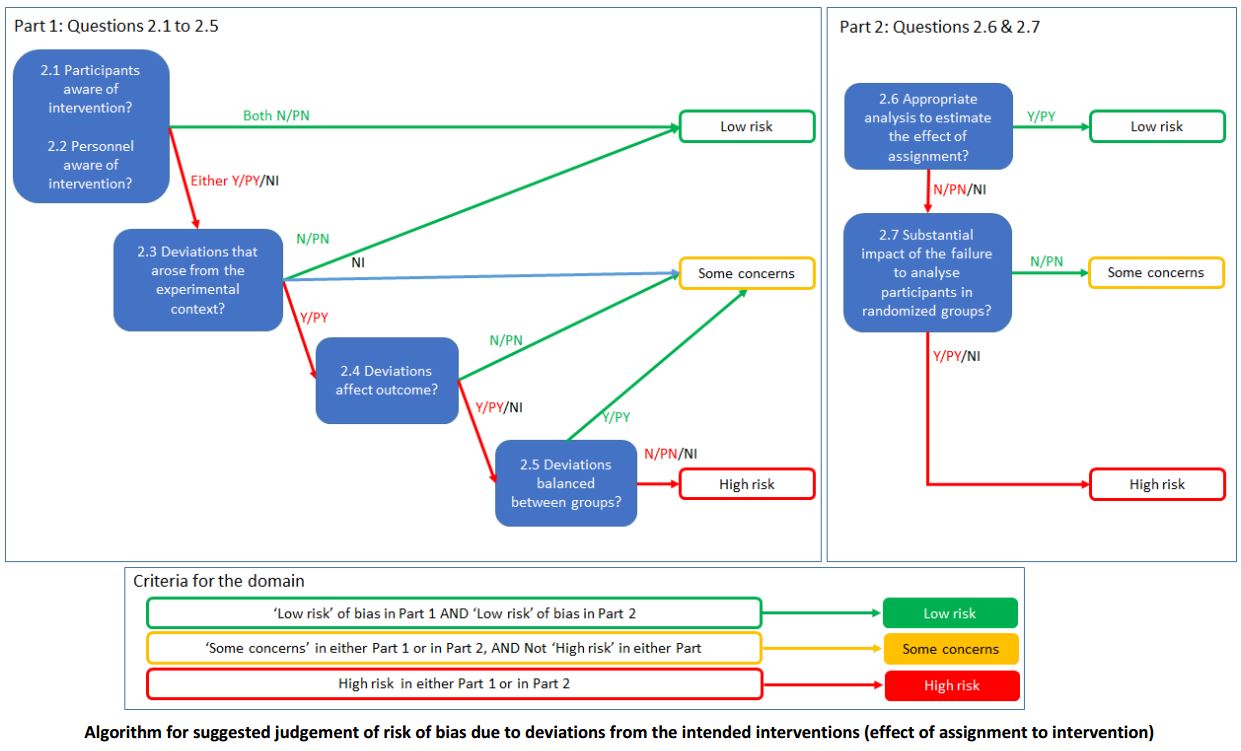


**Domain 2 (b): Risk of bias due to deviations from the intended interventions (effect of adhering to intervention)**2.1 Were participants aware of their assigned intervention during the trial?
2.2 Were carers and people delivering the interventions aware of participants' assigned intervention during the trial?
2.3 If Y/PY/NI to 2.1 or 2.2: Were important co-interventions balanced across intervention groups?
2.4 Could failures in implementing the intervention have affected the outcome?
2.5 Did study participants adhere to the assigned intervention regimen?
2.6 If N/PN/NI to 2.3 or 2.5 or Y/PY/NI to 2.4: Was an appropriate analysis used to estimate the effect of adhering to the intervention?


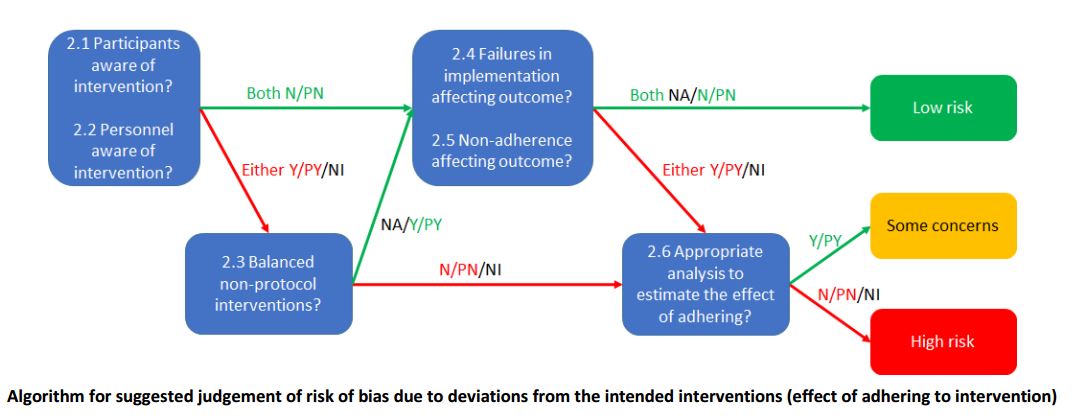


**Domain 3: Missing outcome data**3.1 Were data for this outcome available for all, or nearly all, participants randomized?
3.2 If N/PN/NI to 3.1: Is there evidence that result was not biased by missing outcome data?
3.3 If N/PN to 3.2: Could missingness in the outcome depend on its true value?
3.4 If Y/PY/NI to 3.3: Do the proportions of missing outcome data differ between intervention groups?
3.5 If Y/PY/NI to 3.3: Is it likely that missingness in the outcome depended on its true value?
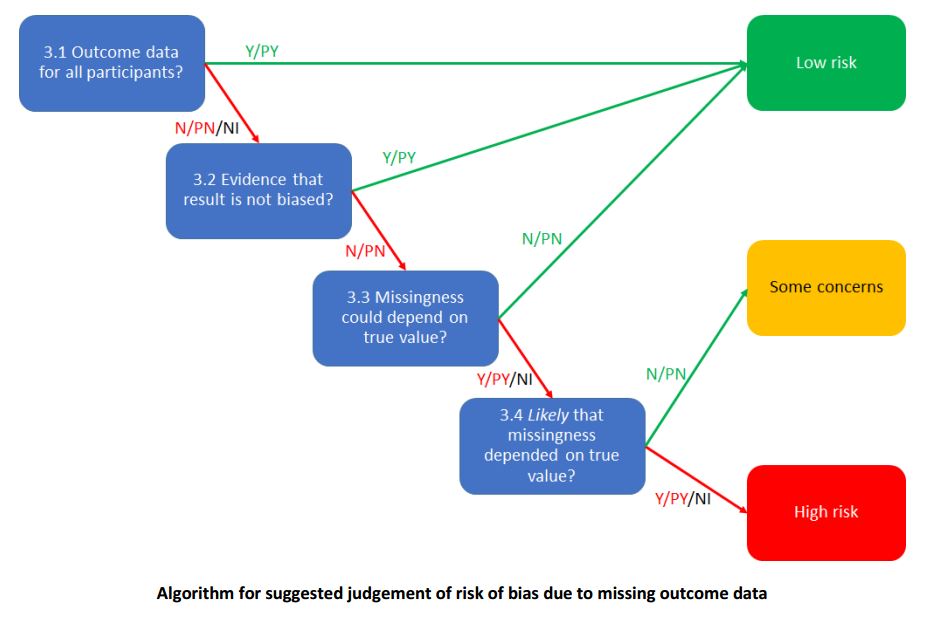


**Domain 4: Risk of bias in measurement of the outcome**4.1 Was the method of measuring the outcome inappropriate?
4.2 Could measurement or ascertainment of the outcome have differed between intervention groups?
4.3 If N/PN/NI to 4.1 and 4.2: Were outcome assessors aware of the intervention received by study participants?
4.4 If Y/PY/NI to 4.3: Could assessment of the outcome have been influenced by knowledge of intervention received?
4.5 If Y/PY/NI to 4.4: Is it likely that assessment of the outcome was influenced by knowledge of intervention received?


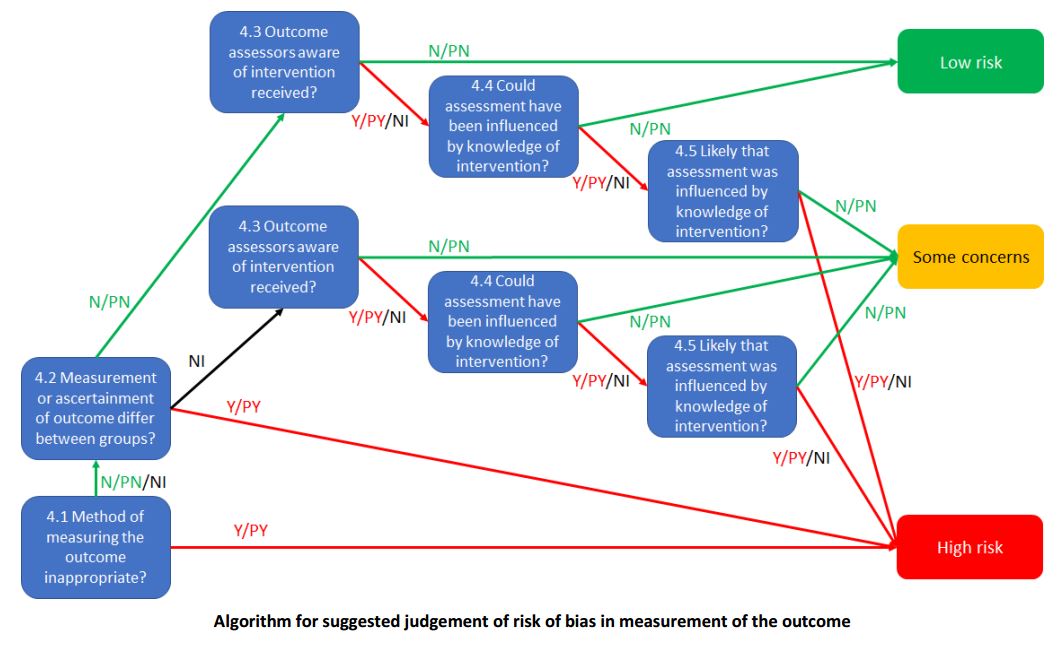


**Domain 5: Risk of bias in selection of the reported result**5.1 Was the trial analysed in accordance with a pre-specified plan that was finalized before unblinded outcome data were available for analysis
Is the numerical result being assessed likely to have been selected, on the basis of the results, from...
5.2 ... multiple outcome measurements (e.g. scales, definitions, time points) within the outcome domain?
5.3 ... multiple analyses of the data?


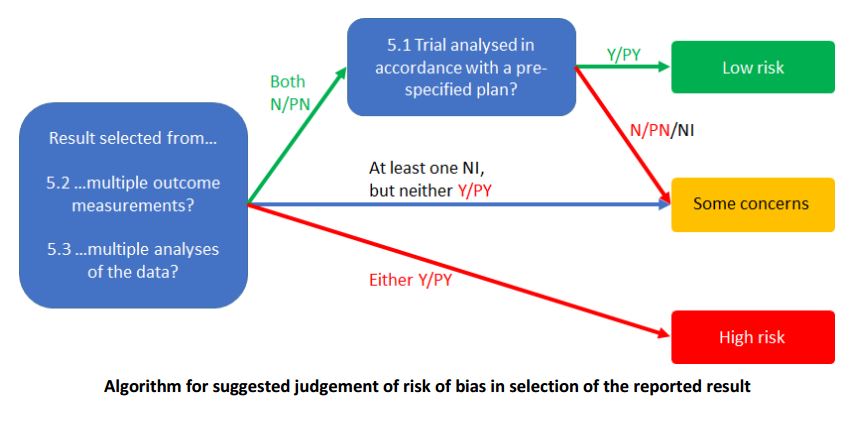


**Appendix 2b. Summary of the ROBINS-I tool [Sterne et al., 2016]**

**Domain 1: Bias due to confounding**1.1 Is there potential for confounding of the effect of intervention in this study?
If N/PN to 1.1: the study can be considered to be at low risk of bias due to confounding and no further signalling questions need be considered
If Y/PY to 1.1: determine whether there is a need to assess time-varying confounding:
1.2 Was the analysis based on splitting participants’ follow up time according to intervention received?
If N/PN, answer questions relating to baseline confounding (1.4 to 1.6)
If Y/PY, go to question 1.3.
1.3 Were intervention discontinuations or switches likely to be related to factors that are prognostic for the outcome?
If N/PN, answer questions relating to baseline confounding (1.4 to 1.6)
If Y/PY, answer questions relating to both baseline and time-varying confounding (1.7 and 1.8)
Questions relating to baseline confounding only
1.4 Did the authors use an appropriate analysis method that controlled for all the important confounding domains?
1.5 If Y/PY to 1.4: Were confounding domains that were controlled for measured validly and reliably by the variables available in this study?
1.6 Did the authors control for any post-intervention variables that could have been affected by the intervention?
Questions relating to baseline and time-varying confounding
1.7 Did the authors use an appropriate analysis method that controlled for all the important confounding domains and for time-varying confounding?
1.8 If Y/PY to 1.7: Were confounding domains that were controlled for measured validly and reliably by the variables available in this study?

**Domain 2: Bias in selection of participants into the study**
2.1 Was selection of participants into the study (or into the analysis) based on participant characteristics
observed after the start of intervention?
If N/PN to 2.1: go to 2.4
2.2 If Y/PY to 2.1: Were the post-intervention variables that influenced selection likely to be associated
with intervention?
2.3 If Y/PY to 2.2: Were the post-intervention variables that influenced selection likely to be influenced by
the outcome or a cause of the outcome?
2.4 Do start of follow-up and start of intervention coincide for most participants?
2.5 If Y/PY to 2.2 and 2.3, or N/PN to 2.4: Were adjustment techniques used that are likely to correct for the presence of selection biases?

**Domain 3: Bias in classification of interventions**3.1 Were intervention groups clearly defined?
3.2 Was the information used to define intervention groups recorded at the start of the intervention?
3.3 Could classification of intervention status have been affected by knowledge of the outcome or risk of the outcome?

**Domain 4: Bias due to deviations from intended interventions**If your aim for this study is to assess the effect of assignment to intervention, answer questions 4.1 and 4.2
4.1 Were there deviations from the intended intervention beyond what would be expected in usual practice?
4.2 If Y/PY to 4.1: Were these deviations from intended intervention unbalanced between groups and likely to have affected the outcome?
If your aim for this study is to assess the effect of starting and adhering to intervention, answer questions 4.3 to 4.6
4. Were important co-interventions balanced across intervention groups?
4.4 Was the intervention implemented successfully for most participants?
4.5 Did study participants adhere to the assigned intervention regimen?
4.6 If N/PN to 4.3, 4.4 or 4.5: Was an appropriate analysis used to estimate the effect of starting and adhering to the intervention?

**Domain 5: Bias due to missing data**5.1 Were outcome data available for all, or nearly all, participants?
5.2 Were participants excluded due to missing data on intervention status?
5.3 Were participants excluded due to missing data on other variables needed for the analysis?
5.4 If PN/N to 5.1, or Y/PY to 5.2 or 5.3: Are the proportion of participants and reasons for missing data similar across interventions?
5.5 If PN/N to 5.1, or Y/PY to 5.2 or 5.3: Is there evidence that results were robust to the presence of missing data?

**Domain 6: Bias in measurement of outcomes**6.1 Could the outcome measure have been influenced by knowledge of the intervention received?
6.2 Were outcome assessors aware of the intervention received by study participants?
6.3 Were the methods of outcome assessment comparable across intervention groups?
6.4 Were any systematic errors in measurement of the outcome related to intervention received?

**Domain 7: Bias in selection of the reported result**Is the reported effect estimate likely to be selected, on the basis of the results, from...
7.1. ... multiple outcome measurements within the outcome domain?
7.2 ... multiple analyses of the intervention-outcome relationship?
7.3 ... different subgroups?


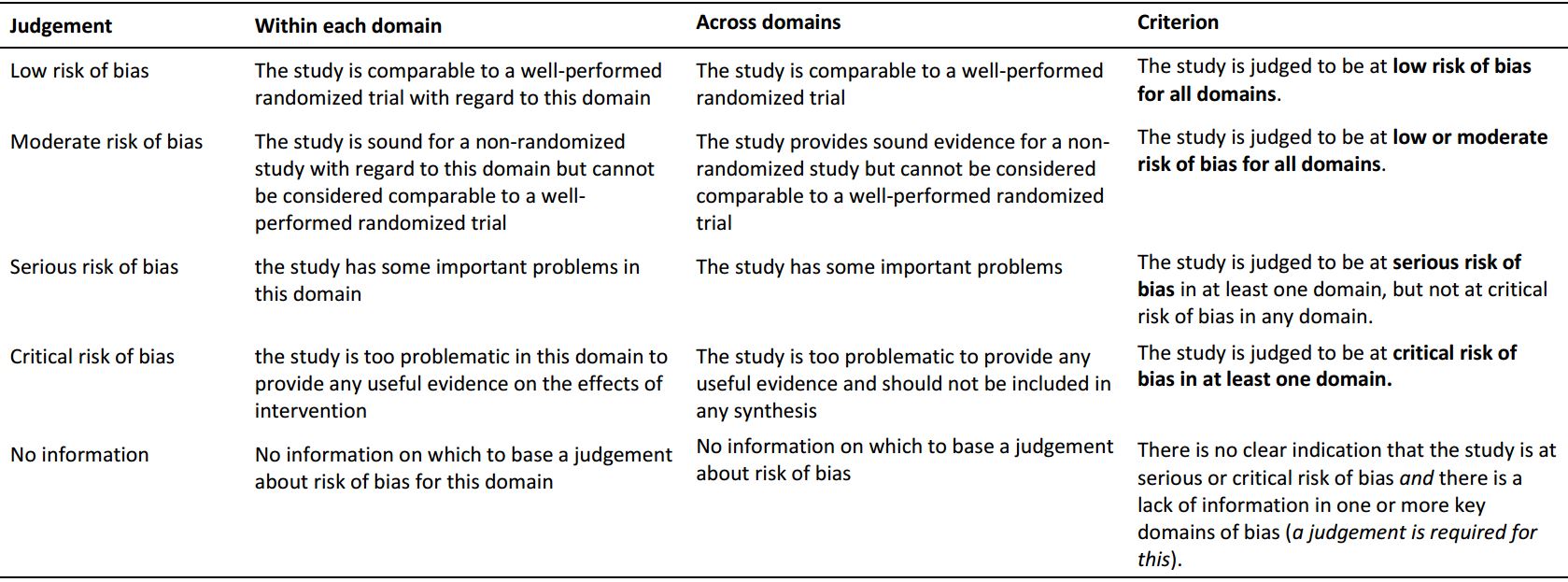


A table for the interpretation of domain-level and overall risk of bias judgments in ROBINS-I

**Appendix 2c. Summary of the ROBIS tool [Whiting et al., 2016]**

**Phase 1: Assessing Relevance (using PICO questions)**

| **Review Type** | Intervention | Aetiology | Diagnosis | Prognosis |
| --- | --- | --- | --- | --- |
| **PICO equivalent** | Patients/population(s)  Intervention(s)  Comparator(s)  Outcome(s) | Patients/population(s)  Exposure(s) and comparator(s)  Outcome(s) | Patients  Index test(s)  Reference standard  Target condition | Patients  Outcome to be predicted  Intended use of model  Intended moment in time |

**Phase 2: Identifying concerns with the review process**

Domain 1: Study eligibility criteria
1.1 Did the review adhere to pre-defined objectives and eligibility criteria?
1.2 Were the eligibility criteria appropriate for the review question?
1.3 Were eligibility criteria unambiguous?
1.4 Were all restrictions in eligibility criteria based on study characteristics appropriate?
1.5 Were any restrictions in eligibility criteria based on sources of information appropriate?

Domain 2: Identification and selection of studies
2.1 Did the search include an appropriate range of databases/ electronic sources for published and unpublished reports?
2.2 Were methods additional to database searching used to identify relevant reports?
2.3 Were the terms and structure of the search strategy likely to retrieve as many eligible studies as possible?
2.4 Were restrictions based on date, publication format, or language appropriate?
2.5 Were efforts made to minimize errors in selection of studies?

Domain 3: Data collection and study appraisal
3.1 Were efforts made to minimize error in data collection?
3.2 Were sufficient study characteristics available for both review authors and readers to be able to interpret the results?
3.3 Were all relevant study results collected for use in the synthesis?
3.4 Was risk of bias (or methodological quality) formally assessed using appropriate criteria?
3.5 Were efforts made to minimize error in risk of bias assessment?

Domain 4: Synthesis and findings
4.1 Did the synthesis include all studies that it should?
4.2 Were all predefined analyses followed or departures explained?
4.3 Was the synthesis appropriate given the nature and similarity in the research questions, study designs and outcomes across included studies?
4.4 Was between-studies variation (heterogeneity) minimal or addressed in the synthesis?
4.5 Were the findings robust, e.g. as demonstrated through funnel plot or sensitivity analyses?
4.6 Were biases in primary studies minimal or addressed in the synthesis?

**Phase 3: Judging risk of bias**

A. Did the interpretation of findings address all of the concerns identified the Phase 2 assessment?
B. Was the relevance of identified studies to the review's research question appropriately considered?
C. Did the reviewers avoid emphasizing results on the basis of their statistical significance?

**Appendix 3: Overall risk of bias assessments for all included studies according to the section, in which they were cited in, in the 2013 German guidelines for the use of fluoride in caries prevention in children, as well as the main conclusion of each paper (n=58).**

| Section in the guideline | Cited paper | Overall risk of bias assessment | Main conclusion |
| --- | --- | --- | --- |
| Fluoride tablets | Hausen et al. 2010 | High | Fluoride lozenges are effective in caries prevention in permanent dentition |
|  | Källestål et al. 2007 | High | No effect for fluoride lozenges compared to fluoride varnish in permanent dentition |
|  | Stecksen-Blicks et al. 2008 | High | No difference between fluoride lozenges or xylitol-fluoride lozenges compared to control group receiving fluoride varnish in permanent dentition |
|  | Haugejorden et al. 2005 | High | More sales of fluoride tablets are associated with improvement in oral health of 5-year-olds |
|  | Momeni et al. 2007 | High | Fluoride tablets are effective in caries prevention in permanent dentition when taken in early childhood |
|  | Wennhall et al. 2008 | High | A combination of toothpaste 1000 ppm and fluoride tablets is effective in caries prevention in young high caries risk children |
|  | Ismail et al. 2008 | Moderate | - Weak evidence supporting fluoride supplements for caries prevention in primary teeth - Some evidence supporting fluoride supplements in permanent dentition |
| Fluoridated toothpastes | Al Jundi et al. 2006 | High | supervised toothbrushing with fluoridated toothpaste is more efficient than non-supervised toothbrushing (children 6- 12 years old) |
|  | Ellwood et al. 2004 | High | No benefit of ‘’free’’ 1450 ppm or 450 ppm toothpastes compared to no ‘’free’’ toothpaste in poor families (children 1 year old at start of the trial) |
|  | Jackson et al. 2005 | Moderate | Supervised toothbrushing with 1450 ppm toothpaste in schools resulted in less caries than no intervention (children 5 – 6 years old) |
|  | Lima et al. 2007 | High | Significant difference between 500 and 1000 ppm toothpaste in high caries risk children but no difference in low caries risk children (2-4 years old) |
|  | Papas et al. 2008 | Low | *Not included in the guideline’s recommendations (considered not relevant by authors of the guidelines)* |
|  | Petersen et al. 2004 | Moderate | *Not included in the guideline’s recommendations (considered not relevant by authors of the guidelines)* |
|  | Pine et al. 2007 | Moderate | Significant difference in caries prevention between supervised toothbrushing with 1000 ppm toothpaste compared to no intervention (children 7 – 12 years old) |
|  | Stookey et al. 2007 | High | No difference between 500 and 1000 ppm toothpaste by children 9 -12 years old |
|  | Do et al. 2007 | High | Using 1000 ppm increases risk of fluorosis without benefit in caries prevention (children 8 – 13) |
|  | Yee et al. 2006 | High | *Not included in the guideline’s recommendations (considered not relevant by authors of the guidelines)* |
|  | Twetman et al. 2003 | Low | - Strong evidence for the use of 1500 ppm instead of 1000 ppm in young permanent dentition. - More benefit with supervised toothbrushing - Incomplete evidence regarding primary dentition |
| Paediatricians’ citations and recommendations | Hamberg 1971 | High | It is recommended to add fluoride to Vitamin D drops for children 0 -3 years old |
|  | Aasenden et al. 1974 | High | Systemic and ingested Fluoride supplements by children 0 - 3 years old are advantageous for the permanent dentition |
|  | Fanning et al. 1975 | High | Only slight benefit of fluoride supplements in the first year of age in caries prevention in primary dentition |
|  | Margolis et al. 1975 | High | Significant difference in caries prevention in favour of fluoride supplements in infants compared to no supplements |
|  | Widenheim et al. 1991 | High | Significant difference in caries prevention in primary dentition in favour of fluoride supplements in pre-school children compared to no supplements |
|  | Tubert-Jeannin et al. 2011 | Low | - Weak evidence on the benefit of fluoride supplements in permanent teeth - Unclear effect of the use of fluoride supplements in primary dentition - No benefit of fluoride supplements compared to topical administration |
|  | Walsh et al. 2010 | Low | - Benefits of using fluoride toothpaste 1000 ppm and above in preventing caries in children and adolescents when compared to placebo - The relative caries preventive effect of fluoride toothpastes of different concentrations increases with higher fluoride concentration - The decision of what fluoride levels to use for children under 6 years should be balanced with the risk of fluorosis |
| Fluoride varnish | Bravo et al. 2005 | High | Better caries protection with sealants compared to fluoride varnish 4 years after discontinuation of the intervention on permanent teeth |
|  | Hardman et al. 2007 | Low | No caries prevention effect of fluoride varnish application twice a year on low caries risk children when used with fluoridated toothpaste (1450 ppm) |
|  | Sköld et al. 2005 I | Moderate | - Fluoride varnish at least twice a year shows a caries prevention effect in adolescents 13-16 years old, especially in moderate- and high caries risk populations - Application 8 times a year showed more caries prevention effect than 2 or 3 times a year |
|  | Stecksén-Blicks et al. 2007 | Low | Fluoride varnish reduces initial caries lesions in adolescents with fixed orthodontic appliances |
|  | Weintraub et al. 2006 | High | Less caries incidence with increased number of applications of fluoride varnish in pre-school children |
|  | Borutta et al. 2006 | Moderate | Effect in caries reduction using fluoride varnish in pre-school children compared to no intervention |
|  | Ibricevic et al. 2005 | High | Effect in caries reduction using fluoride varnish (twice yearly) on children with special needs (moderate caries risk), but no effect on low caries risk children |
|  | Vivaldi-Rodriguez et al. 2006 | High | Fluoride varnish 4 times a year is effective in preventing the development of white spot lesions in adolescents with fixed orthodontic appliances |
|  | Xhemnica et al. 2008 | High | *Not included in the guideline’s recommendations (considered not relevant by authors of the guidelines)* |
|  | ADA Council on Scientific Affairs 2006 | Moderate | Periodic fluoride treatments should be considered for children and adults at moderate or high caries risk |
|  | Azarpazhooh et al. 2008 | Moderate | Fluoride varnish should be applied twice a year for high caries risk population |
|  | Derks et al. 2004 | High | The use of a polymeric tooth coating on the tooth surface around brackets showed almost no demineralization-inhibiting effect |
|  | Petersson et al. 2004 | Low | There is only limited evidence for the caries preventive effect of topical application of fluoride varnish in permanent teeth |
| Fluoridated mouthwash | Øgaard et al. 2006 | Moderate | A slightly more benefit in caries reduction using amine fluoride/stannous fluoride toothpaste/mouth rinse combination compared to combinations containing sodium fluoride in orthodontic patients |
|  | Sköld et al. 2005 | Moderate | School-based fluoride mouth rinsing, as a supplement to the daily use of fluoridated toothpaste reduces caries incidence on approximal surfaces in adolescents with low to moderate caries risk |
|  | Willmot et al. 2004 | High | No difference in white lesions reduction in orthodontic patients between using fluoride-free toothpaste with a fluoridated mouthwash compared to only fluoride-free toothpaste |
|  | Wyatt et al. 2004 | Moderate | 0.2% neutral NaF mouth rinse every day reduces the incidence of caries among elders in care facilities |
|  | Pieterse et al. 2006 | High | Long-term rinsing with fluoride (> 3 years) has a positive effect on teeth status |
|  | Benson et al. 2005 | Low | There is some evidence that the use of a daily NaF mouth rinse or a GIC for bonding brackets might reduce the occurrence and severity of white spot lesions during orthodontic treatment |
|  | Benson et al. 2008 | Low | Same as Benson et al. 2005 |
|  | Marinho et al. 2004a | Low | There is no clear suggestion that fluoride varnish is more effective than mouth rinses and the evidence for the comparative effectiveness of fluoride varnishes with gels, and mouth rinses with gels is inconclusive |
|  | Twetman et al. 2004 | Low | Sodium fluoride mouth rinses may have an anti-caries effect in children with limited background of fluoride exposure, while its additional effect along with the daily use of fluoridated toothpaste could be questioned |
| Fluoride gel | Ersin et al. 2008 | High | No significant difference in caries increment between patients in 3 groups with: chlorhexidine varnish, sodium fluoride gel (4000 ppm) or dental health education (all groups used 1500 ppm fluoridated toothpaste) |
|  | Jiang et al. 2005 | Low | Bi-annual professional application of acidulated phosphate fluoride foam was effective in reducing (approximal) caries increment in primary teeth |
|  | Karlsson et al. 2007 | High | Adjunctive weekly brushing of amine fluoride gel achieved no significant enhancement of remineralization of white spot lesions monitored with the quantitative light-induced fluorescence method |
|  | Truin et al. 2005a | Low | The treatment effect of fluoride gel application (4500 ppm) on dentinal caries lesions in low caries risk children was considered not clinically relevant |
|  | Truin et al. 2005b | Low | Professionally applied fluoride gel (4500 ppm) showed no statistically significant effect on mean D_3_MFS score in low caries risk 9.5 to 11.5-year-olds |
|  | Truin et al. 2007 | Low | Professionally applied fluoride gel (4500 ppm) showed no statistically significant caries-inhibiting effect on both enamel and dentine lesions in the permanent dentition of low caries children |
|  | Van Rijkom et al. 2004 | Low | In the investigated low caries risk 4.5 to 6.5-year-olds, fluoride gel treatment (4500 ppm) showed a statistically significant (tested one-sided) caries-inhibiting effect on D_3_MFS, which was considered not clinically relevant, with no significant effect on d3mfs |
| Systematic reviews | Ammari et al. 2007 | Low | Fluoride-based interventions appear to be effective in young children |
|  | Australian National Health and Medical Research Council 2007 | High | Fluoridation of drinking water remains the most effective and socially equitable means of achieving community-wide exposure to the caries prevention effects of fluoride |
|  | Hiiri et al. 2006 | Low | Some evidence of the superiority of pit and fissure sealants over fluoride varnish application in the prevention of occlusal decays. However, it remained unclear to what extent there is difference between the effectiveness of pit and fissure sealants and fluoride varnishes |
|  | Marinho et al. 2004b | Low | Topical fluorides (mouth rinses, gels, or varnishes) used in addition to fluoride toothpaste achieve a modest reduction in caries compared to toothpaste used alone. No conclusions about any adverse effects could be reached |

*References of appendix 3 (in alphabetical order):

- Aasenden R, Peebles TC. Effects of fluoride supplementation from birth on human deciduous and permanent teeth. Arch Oral Biol. 1974;19(4):321-6. doi: 10.1016/0003-9969(74)90194-0.
- Al-Jundi SH, Hammad M, Alwaeli H. The efficacy of a school-based caries preventive program: a 4-year study. Int J Dent Hyg. 2006;4(1):30-4. doi: 10.1111/j.1601-5037.2006.00156.x.
- American Dental Association Council on Scientific A. Professionally applied topical fluoride: evidence-based clinical recommendations. J Am Dent Assoc. 2006;137(8):1151-9. doi: 10.14219/jada.archive.2006.0356.
- Ammari JB, Baqain ZH, Ashley PF. Effects of programs for prevention of early childhood caries. A systematic review. Med Princ Pract. 2007;16(6):437-42. doi: 10.1159/000107748
- Australian National Health and Medical Research Council 2007. Yeung CA. A systematic review of the efficacy and safety of fluoridation. Evid Based Dent. 2008;9(2):39-43. doi: 10.1038/sj.ebd.6400578.
- Azarpazhooh A, Main PA. Fluoride varnish in the prevention of dental caries in children and adolescents: a systematic review. J Can Dent Assoc. 2008;74(1):73-9.
- Benson PE, Parkin N, Millett DT, Dyer FE, Vine S, Shah A. Fluorides for the prevention of white spots on teeth during fixed brace treatment. Cochrane Database Syst Rev. 2004(3):CD003809. doi: 10.1002/14651858.CD003809.pub2
- Benson PE, Shah AA, Millett DT, Dyer F, Parkin N, Vine RS. Fluorides, orthodontics and demineralization: a systematic review. J Orthod. 2005;32(2):102-14. doi: 10.1179/146531205225021033.
- Borutta A, Reuscher G, Hufnagl S, Mobius S. Kariesprophylaxe mit Fluoridlacken bei Vorschulkindern [Caries prevention with fluoride varnishes among preschool children]. Gesundheitswesen. 2006;68(11):731-4.
- Bravo M, Montero J, Bravo JJ, Baca P, Llodra JC. Sealant and fluoride varnish in caries: a randomized trial. J Dent Res. 2005;84(12):1138-43. doi: 10.1177/154405910508401209.
- Derks A, Katsaros C, Frencken JE, van't Hof MA, Kuijpers-Jagtman AM. Caries-inhibiting effect of preventive measures during orthodontic treatment with fixed appliances. A systematic review. Caries Res. 2004;38(5):413-20. doi: 10.1159/000079621.
- Do LG, Spencer AJ. Risk-benefit balance in the use of fluoride among young children. J Dent Res. 2007;86(8):723-8. doi: 10.1177/154405910708600807.
- Ellwood RP, Davies GM, Worthington HV, Blinkhorn AS, Taylor GO, Davies RM. Relationship between area deprivation and the anticaries benefit of an oral health programme providing free fluoride toothpaste to young children. Community Dent Oral Epidemiol. 2004;32(3):159-65. doi: 10.1111/j.1600-0528.2004.00150.x.
- Ersin NK, Eden E, Eronat N, Totu FI, Ates M. Effectiveness of 2-year application of school-based chlorhexidine varnish, sodium fluoride gel, and dental health education programs in high-risk adolescents. Quintessence international (Berlin, Germany : 1985). 2008;39(2):e45-51.
- Fanning EA, Cellier KM, Leadbeater MM, Somerville CM. South Australian kindergarten children: fluoride tablet supplements and dental caries. Aust Dent J. 1975;20(1):7-9. doi: 10.1111/j.1834-7819.1975.tb03507.x.
- Hamberg L. Controlled trial of fluoride in vitamin drops for prevention of caries in children. Lancet. 1971;1(7696):441-2. doi: 10.1016/s0140-6736(71)92426-3.
- Hardman MC, Davies GM, Duxbury JT, Davies RM. A cluster randomised controlled trial to evaluate the effectiveness of fluoride varnish as a public health measure to reduce caries in children. Caries Res. 2007;41(5):371-6. doi: 10.1159/000104795.
- Haugejorden O, Klock KS, Trovik TA. Developing a scale for measuring expectancy of retaining natural teeth for life and comparison of results obtained using a global item and a multi-item scale of measurement. Acta Odontol Scand. 2005;63(1):36-42. doi: 10.1080/00016350510019711.
- Hausen H, Seppa L, Poutanen R, Niinimaa A, Lahti S, Karkkainen S, et al. Noninvasive control of dental caries in children with active initial lesions. A randomized clinical trial. Caries Res. 2007;41(5):384-91. doi: 10.1159/000104797.
- Hiiri A, Ahovuo-Saloranta A, Nordblad A, Makela M. Pit and fissure sealants versus fluoride varnishes for preventing dental decay in children and adolescents. Cochrane Database Syst Rev. 2006(4):CD003067. doi: 10.1002/14651858.CD003067.pub2
- Ibricevic A, Pekosz A, Walter MJ, Newby C, Battaile JT, Brown EG, et al. Influenza virus receptor specificity and cell tropism in mouse and human airway epithelial cells. J Virol. 2006;80(15):7469-80. doi: 10.1128/JVI.02677-05.
- Ismail AI, Hasson H. Fluoride supplements, dental caries and fluorosis: a systematic review. J Am Dent Assoc. 2008;139(11):1457-68. doi: 10.14219/jada.archive.2008.0071.
- Jackson RJ, Newman HN, Smart GJ, Stokes E, Hogan JI, Brown C, et al. The effects of a supervised toothbrushing programme on the caries increment of primary school children, initially aged 5-6 years. Caries Res. 2005;39(2):108-15. doi: 10.1159/000083155.
- Jiang H, Bian Z, Tai BJ, Du MQ, Peng B. The effect of a bi-annual professional application of APF foam on dental caries increment in primary teeth: 24-month clinical trial. J Dent Res. 2005;84(3):265-8. doi: 10.1177/154405910508400311.
- Kallestal C, Fjelddahl A. A four-year cohort study of caries and its risk factors in adolescents with high and low risk at baseline. Swed Dent J. 2007;31(1):11-25.
- Karlsson L, Lindgren LE, Trollsas K, Angmar-Mansson B, Tranaeus S. Effect of supplementary amine fluoride gel in caries-active adolescents. A clinical QLF study. Acta Odontol Scand. 2007;65(5):284-91. doi: 10.1080/00016350701620644.
- Lima TJ, Ribeiro CC, Tenuta LM, Cury JA. Low-fluoride dentifrice and caries lesion control in children with different caries experience: a randomized clinical trial. Caries Res. 2008;42(1):46-50. doi: 10.1159/000111749.
- Margolis FJ, Reames HR, Freshman E, MaCauley CD, Mehaffey H. Flouride. Ten-year prospective study of deciduous and permanent dentition. Am J Dis Child. 1975;129(7):794-800. doi: 10.1001/archpedi.1975.02120440020006.
- Marinho VC, Higgins JP, Sheiham A, Logan S. Combinations of topical fluoride (toothpastes, mouthrinses, gels, varnishes) versus single topical fluoride for preventing dental caries in children and adolescents. Cochrane Database Syst Rev. 2004a;2004(1):CD002781. doi: 10.1002/14651858.CD002781.pub2.
- Marinho VC, Higgins JP, Sheiham A, Logan S. One topical fluoride (toothpastes, or mouthrinses, or gels, or varnishes) versus another for preventing dental caries in children and adolescents. Cochrane Database Syst Rev. 2004b;2004(1):CD002780. doi: 10.1002/14651858.CD002780.pub2.
- Momeni A, Hartmann T, Born C, Heinzel-Gutenbrunner M, Pieper K. Association of caries experience in adolescents with different preventive measures. Int J Public Health. 2007;52(6):393-401. doi: 10.1007/s00038-007-6094-x.
- Ogaard B, Alm AA, Larsson E, Adolfsson U. A prospective, randomized clinical study on the effects of an amine fluoride/stannous fluoride toothpaste/mouthrinse on plaque, gingivitis and initial caries lesion development in orthodontic patients. Eur J Orthod. 2006;28(1):8-12. doi: 10.1093/ejo/cji075.
- Papas A, Russell D, Singh M, Kent R, Triol C, Winston A. Caries clinical trial of a remineralising toothpaste in radiation patients. Gerodontology. 2008;25(2):76-88. doi: 10.1111/j.1741-2358.2007.00199.x.
- Petersen PE, Peng B, Tai B, Bian Z, Fan M. Effect of a school-based oral health education programme in Wuhan City, Peoples Republic of China. Int Dent J. 2004;54(1):33-41. doi: 10.1111/j.1875-595x.2004.tb00250.x.
- Petersson LG, Twetman S, Dahlgren H, Norlund A, Holm AK, Nordenram G, et al. Professional fluoride varnish treatment for caries control: a systematic review of clinical trials. Acta Odontol Scand. 2004;62(3):170-6. doi: 10.1080/00016350410006392.
- Pieterse S, de Jong N, de Vos N. Does fluoride rinsing have an effect on teeth status? Evaluation of preventive dental health activities for the youth of Woudenberg, The Netherlands. Int J Dent Hyg. 2006;4(3):133-9. doi: 10.1111/j.1601-5037.2006.00183.x.
- Pine CM, Curnow MM, Burnside G, Nicholson JA, Roberts AJ. Caries prevalence four years after the end of a randomised controlled trial. Caries Res. 2007;41(6):431-6. doi: 10.1159/000104800.
- Skold MU, Birkhed D, Borg E, Petersson LG. Approximal caries development in adolescents with low to moderate caries risk after different 3-year school-based supervised fluoride mouth rinsing programmes. Caries Res. 2005;39(6):529-35. doi: 10.1159/000088191.
- Skold MU, Petersson LG, Lith A, Birkhed D. Effect of school-based fluoride varnish programmes on approximal caries in adolescents from different caries risk areas. Caries Res. 2005;39(4):273-9. doi: 10.1159/000084833.
- Stecksen-Blicks C, Holgerson PL, Twetman S. Effect of xylitol and xylitol-fluoride lozenges on approximal caries development in high-caries-risk children. Int J Paediatr Dent. 2008;18(3):170-7. doi: 10.1111/j.1365-263X.2007.00912.x.
- Stecksen-Blicks C, Renfors G, Oscarson ND, Bergstrand F, Twetman S. Caries-preventive effectiveness of a fluoride varnish: a randomized controlled trial in adolescents with fixed orthodontic appliances. Caries Res. 2007;41(6):455-9. doi: 10.1159/000107932.
- Stookey GK, Mau MS, Isaacs RL, Gonzalez-Gierbolini C, Bartizek RD, Biesbrock AR. The relative anticaries effectiveness of three fluoride-containing dentifrices in Puerto Rico. Caries Res. 2004;38(6):542-50. doi: 10.1159/000080584.
- Truin GJ, van 't Hof MA. Professionally applied fluoride gel in low-caries 10.5-year-olds. J Dent Res. 2005b;84(5):418-21. doi: 10.1177/154405910508400504.
- Truin GJ, van't Hof M. The effect of fluoride gel on incipient carious lesions in a low-caries child population. Community Dent Oral Epidemiol. 2007;35(4):250-4. doi: 10.1111/j.1600-0528.2007.00333.x.
- Truin GJ, van't Hof MA. Caries prevention by professional fluoride gel application on enamel and dentinal lesions in low-caries children. Caries Res. 2005a;39(3):236-40. doi: 10.1159/000084804.
- Tubert-Jeannin S, Auclair C, Amsallem E, Tramini P, Gerbaud L, Ruffieux C, et al. Fluoride supplements (tablets, drops, lozenges or chewing gums) for preventing dental caries in children. Cochrane Database Syst Rev. 2011;2011(12):CD007592. doi: 10.1002/14651858.CD007592.pub2.
- Twetman S, Axelsson S, Dahlgren H, Holm AK, Kallestal C, Lagerlof F, et al. Caries-preventive effect of fluoride toothpaste: a systematic review. Acta Odontol Scand. 2003;61(6):347-55. doi: 10.1080/00016350310007590.
- van Rijkom HM, Truin GJ, van 't Hof MA. Caries-inhibiting effect of professional fluoride gel application in low-caries children initially aged 4.5-6.5 years. Caries Res. 2004;38(2):115-23. doi: 10.1159/000075935.
- Vivaldi-Rodrigues G, Demito CF, Bowman SJ, Ramos AL. The effectiveness of a fluoride varnish in preventing the development of white spot lesions. World J Orthod. 2006;7(2):138-44.
- Walsh T, Worthington HV, Glenny AM, Appelbe P, Marinho VC, Shi X. Fluoride toothpastes of different concentrations for preventing dental caries in children and adolescents. Cochrane Database Syst Rev. 2010(1):CD007868. doi: 10.1002/14651858.CD007868.pub2.
- Weintraub JA, Ramos-Gomez F, Jue B, Shain S, Hoover CI, Featherstone JD, et al. Fluoride varnish efficacy in preventing early childhood caries. J Dent Res. 2006;85(2):172-6. doi: 10.1177/154405910608500211.
- Wennhall I, Matsson L, Schroder U, Twetman S. Outcome of an oral health outreach programme for preschool children in a low socioeconomic multicultural area. Int J Paediatr Dent. 2008;18(2):84-90. doi: 10.1111/j.1365-263X.2007.00903.x.
- Widenheim J, Birkhed D. Caries-preventive effect on primary and permanent teeth and cost-effectiveness of an NaF tablet preschool program. Community Dent Oral Epidemiol. 1991;19(2):88-92. doi: 10.1111/j.1600-0528.1991.tb00117.x.
- Willmot DR. White lesions after orthodontic treatment: does low fluoride make a difference? J Orthod. 2004;31(3):235-42; discussion 02. doi: 10.1179/146531204225022443.
- Wyatt CC, MacEntee MI. Caries management for institutionalized elders using fluoride and chlorhexidine mouthrinses. Community Dent Oral Epidemiol. 2004;32(5):322-8. doi: 10.1111/j.1600-0528.2004.00176.x.
- Xhemnica L, Sulo D, Rroco R, Hysi D. Fluoride varnish application: a new prophylactic method in Albania. Effect on enamel carious lesions in permanent dentition. Eur J Paediatr Dent. 2008;9(2):93-6.
- Yee R, McDonald N, Helderman WH. Gains in oral health and improved quality of life of 12-13-year-old Nepali schoolchildren: outcomes of an advocacy project to fluoridate toothpaste. Int Dent J. 2006;56(4):196-202. doi: 10.1111/j.1875-595x.2006.tb00094.x.

Appendix 4: Assessment of the recommendations and statements of the German guidelines when considering the risk of bias of underlying literature in the different sections

| Section in the guidelines | Original recommendation | Considering risk of bias of underlying literature |
| --- | --- | --- |
| Fluoride tablets | Fluoride tablets have a topical as well as a systemic effect in caries prevention, with more benefit in the topical application on the long-term. Recommendation: Fluoride lozenges are recommended after teeth eruption | This is not supported with cited evidence of low or moderate risk of bias |
|  | Fluoride tablets should not be used, if fluoridated salt is used regularly at home. | This is not supported with cited evidence of low or moderate risk of bias |
|  | The evidence for the effect of fluoride tablets is more convincing for primary dentition than the permanent dentition | - Weak evidence supporting fluoride supplements for caries prevention in primary teeth - Some evidence supporting fluoride supplements in permanent dentition |
|  | Fluoride tablets are not recommended during pregnancy, due to lack of evidence | No evidence supporting the benefit of fluoride supplementation prenatally |
| Fluoridated toothpastes | - The use of fluoridated toothpaste 500 ppm is recommended from eruption of first primary tooth until the age of 2 in smear amount - Starting from the second year of age, a pea-size amount is recommended | This is not supported with evidence of low or moderate risk of bias |
|  | With the eruption of the first permanent tooth, the use of fluoridated toothpaste 1000 ppm is recommended | This is not supported with cited evidence of low or moderate risk of bias The evidence with low risk of bias suggests 1500 ppm |
|  | - The caries prevention effect increases with increased concentration of fluoride - Toothpastes with fluoride concentration > 1000 ppm are not recommended for pre-school children | This is not supported with cited evidence of low or moderate risk of bias |
|  | - The caries prevention effect increases with the frequency of toothbrushing and with supervision from adults - Toothbrushing until the age of 2 only once a day, but starting from the age of 2 twice daily | More benefit with supervised toothbrushing compared to no intervention   - using 1000 ppm fluoridated toothpaste in pre-school children - using 1450 ppm fluoridated toothpaste in children (7 – 12 years old) |
|  | Fluoride tablets should not be used, if fluoridated salt and fluoridated toothpastes are used regularly at home | This cannot be supported with cited evidence of low or moderate risk of bias |
| Paediatricians’ citations and recommendations | The use of fluoridated toothpaste is not recommended for pre-school children unless they can spit out all toothpaste from the mouth | This cannot be supported with cited evidence of low or moderate risk of bias |
|  | The use of fluoridated toothpaste 450 – 550 ppm has no effect in caries reduction and should not be used | Benefits of using fluoride toothpaste 1000 ppm and above in preventing caries in children and adolescents when compared to placebo |
|  | The use of fluoridated toothpaste 1000 ppm and more is not recommended for pre-school children due to the risk of fluorosis | - This cannot be supported with cited evidence of low or moderate risk of bias - Cited evidence with low or moderate risk of bias states: the decision of what fluoride levels to use for children under 6 years should be balanced with the risk of fluorosis |
|  | The use of fluoride supplements (tablets, lozenges etc..) is recommended for pre-school children | - This cannot be supported with cited evidence of low or moderate risk of bias - Cited evidence with low or moderate risk of bias states: - Weak evidence on the benefit of fluoride supplements in permanent teeth - Unclear effect of the use of fluoride supplements in primary dentition - No benefit of fluoride supplements compared to topical administration |
| Fluoride varnish | The use of fluoride varnish on children has no side effects | No cited literature in this section can support this statement |
|  | Fluoride varnish application is recommended for children and adolescents twice a year regardless of other caries preventive fluoridation measurements | Fluoride varnish application may not be necessary for low caries risk population, but is recommended for moderate and high caries risk children and adolescents, and when orthodontic appliances are fixed in the mouth |
|  | The frequency of applications of the fluoride varnish should increase with increased caries risk (4x a year) | Application of fluoride varnish 8 times a year showed more caries prevention than 2 or 3 times a year in moderate and high caries risk population |
| Fluoridated mouthwash | Supervised daily use of fluoridated mouthwash (0,05% NaF) or weekly use of fluoridated mouthwash (0,2% NaF) shows a caries prevention effect and is therefore recommended for children older than 6 years old and adolescents especially for those with fixed orthodontics or high caries risk | No recommendation for the use of fluoridated mouthwash for caries prevention as an alternative to fluoridated toothpaste or to fluoride varnish, but could be beneficial as a supplement, especially in orthodontic patients |
| Fluoride gel | The use of fluoride gel is recommended regardless of the use of fluoridated toothpaste or other fluoridation supplements | No cited literature in this section can support this statement |
|  | The effect of fluoride gels in caries prevention is not related to the method of its application and can be individually chosen | No cited literature in this section can support this statement |
|  | The frequency of the use of fluoride gel may be increased by patients with active caries, because the caries prevention effect correlates with frequency of the use | No cited literature in this section can support this statement |
| Systematic reviews | The reviews were listed without clear recommendation for clinicians | |
